# Edinburgh Professors

**Published:** 1843-09

**Authors:** 


*Edinburgh Professors.*—An evil is complained of at Edinburgh as contributing yearly to diminish the number of graduates at that university. It is said to be perfectly evident to all who are attending in the Scottish capital that of two of the professors, the suns, although they blazed for a long series of years with much brightness, are now near their western horizon. The fact is mentioned in Edinburgh without any invidious feeling, because there are two parties always to be considered on these occasions, the professors and *the students*, —the teachers and *the taught.* Dr. Hope has long been one of the ornaments of chemistry in this country, and his discoveries most valuable; but his powers of lecturing and distinct articulation now fail him; and although his name will be remembered as long as science exists, his ability as a *lecturer* is now nearly extinct. Dr. Monro, too, has also lost his power of demonstrating, and has of late years acquired a manner of lecturing which is considered to have had a very depreciating effect on the attendance at Edinburgh. It is an object of serious inconvenience at any university when it has not at its disposal a retiring fund for professors. It is not desirable that such men as Drs. Hope and Monro should be exposed to the consequences which in their classes they have experienced during the last two or three years. Those professors have, in fact, no longer any command over them. As to the students who now avoid the university,—the Irish students, more especially, have gone to other universities—on inquiring the reason of any one who is acquainted with the fact, the reply is, "It is because the classes of Drs. Hope and Monro are imperative on students graduating, for it was the cheapness of college classes, the small expense of living in Edinburgh, and the fame of the professors, all combined, that rendered the university so desirable a place for many of the pupils. Amonst the students, generally, the remedy is considered to consist either in Drs. Hope and Monro retiring, or for the senatus to declare that their lectures shall no longer be compulsory."—*Ibid.*

